# Angela Nguku: for a societal approach to primary health care

**DOI:** 10.2471/BLT.20.031120

**Published:** 2020-11-01

## Abstract

Angela Nguku talks to Gary Humphreys about the vital importance of community involvement in primary health care.

Q: You received your Bachelor’s in nursing and midwifery, then went on to a Master’s in project planning and management. What inspired that change of direction?

A: For me it wasn’t a change of direction. I studied nursing and midwifery because I wanted to help people. I studied management for the same reason. But to be honest there were things about being a nurse within the government health system that left me uncomfortable.

Q: Like what?

A: The caps for one thing (laughing)! Each cadre of nurses was identified by a colour code on the caps. It was a hierarchical thing and it got in the way of teamwork which I consider to be vital to effective nursing care. Also, I thought it was emblematic of a colonial system geared more towards maintaining the status quo than meeting the needs of the patients. I had serious doubts about being able to fit into such a system.

Q: Is that what led to you to apply for a job with the AAH-I (Action Africa Help International) – the chance to get out of the city and the public health system?

A: I was certainly aware of AAH-I’s commitment to helping vulnerable people in remote communities and knew I might be sent to some interesting places.

“There is an urgent need to enhance community health literacy.”

Q: Where did they send you?

A: To South Sudan. I was flown in on a single prop plane and left in the middle of nowhere. This was in 2005, two years after the end of the civil war. It was the first time I had been in a war-torn country and it was quite a shock. I almost got back on the plane!

Q: What were you sent to do?

A: Needs assessment for a planned maternal health initiative. The conditions I encountered were shocking. There was no antenatal care, no blood banks, no ambulances, dilapidated facilities that were without equipment or medicines or electricity. Women were giving birth at home and only coming into the health facilities as a last resort because they knew how bad they were. I saw a lot of terrible things during my time there, but one experience really stuck with me. It was seeing this man standing beside his wife’s grave. He had taken his wife to the clinic, but she had died giving birth to triplets. The babies were buried next to her. Seeing that man’s grief, knowing that the deaths had probably been preventable, really made an impression on me. I decided to dedicate myself to doing something about it.

Q: What did you do?

A: I stayed on in South Sudan for two years, training and mentoring the semi-skilled birth attendants and auxiliary midwives that were working in the community, sharing my knowledge about best practice, including the importance of antenatal examinations, and helping them improve their skills in managing obstetric emergencies. I also helped them to develop referral practices where hospital care was accessible. Later on, working with Amref Health Africa, I set up the first training school for midwives in the country at the National Health Training Institute in Maridi County. So, there was all this capacity-building work, but I also spent a lot of time in the communities. I developed a strong sense of the importance of empowering people through health literacy, including education about basic prevention and control of disease, aspects of primary health care (PHC) that tend to be neglected in the prevailing medical model.

Q: What do you mean?

A: I mean the model that puts the emphasis on curative care dispensed by the health system per se – rather than viewing PHC as a broader societal issue. I think it’s important to remember that the PHC concept as framed by the Alma-Ata Declaration includes eight components, the first three of which relate to conditions outside the health system per se, namely: health education on prevailing health problems and the methods of preventing and controlling them; nutritional promotion including food supply; and supply of adequate safe water and sanitation. The Alma-Ata emphasis on health education and, in particular, education about methods of preventing and controlling disease has never seemed more relevant than in the context of the current pandemic.

Q: Can you say more about that?

A: If there is one important lesson the Covid-19 pandemic is teaching us, it is that there is an urgent need to enhance community health literacy as a part of promoting greater self-reliance. A better understanding of disease transmission not only makes people more likely to take the necessary preventive steps, it also increases receptivity to government guidance and helps people navigate the misinformation and rumours with which they are bombarded on a daily basis. In Kenya, the government regularly issues guidance regarding preventive measures such as social distancing, mask wearing and handwashing with soap or hand sanitisers. However – and despite the government’s emphasis on the need for personal responsibility and community engagement – this critical life-saving message falls on deaf ears. You have to ask yourself why.

Q: Is lack of health literacy the answer to that question?

A: It is part of the answer. Because another key issue is over-reliance on government – seeing the pandemic as an issue that must be dealt with by the government rather than by the individual. The two issues are related of course: arming people with the relevant knowledge and skills places individuals, families and communities on a path of self-reliance and is key to advancing the PHC and broader UHC (universal health coverage) agenda.

Q: But self-reliance only takes you so far; you still need a functioning health system, don’t you?

A: Of course, you do. And here at the White Ribbon Alliance (WRA) Kenya we are doing our utmost to ensure that the government follows through on the commitments it has made to providing UHC.

Q: Can you tell us a little about the White Ribbon Alliance?

A: It’s a global, people-led alliance that was formed in 1999 to give a voice to women who were not being heard. Since then, the alliance has spread around the world. I founded the Kenya alliance in 2009 but didn’t decide to concentrate all my efforts on it until 2017, which is when it really took off here.

Q: What are your main areas of activity?

A: WRA Kenya is currently involved in several campaigns to raise awareness of the results from the What Women Want (WWW) campaign, which elicited responses from 1.2 million women worldwide about what they want from health care rather than what health policy experts might think is important. With specific regard to UHC, last year we launched a UHC for Me project that is working to ensure the country’s slowly developing UHC system reflects and responds to community self-articulated needs, prioritizing the most vulnerable and marginalized populations, such as adolescent girls and people with disabilities. This is in addition to other projects that build on action agendas derived from the WWW campaign results.

“We could achieve so much more […] by following the Alma-Ata guidance.”

Q: You say, ‘slowly developing UHC system’. Are you concerned about the pace of UHC-oriented reform?

A: Yes. Unfortunately, like other high-profile global health agendas, UHC is something that gets talked about a lot but doesn’t necessarily result in change on the ground. And I don’t just mean in Kenya. I have seen the same dynamic at work in a number of countries, having headed up the Action2020 sexual and reproductive health and rights (SRHR) and family planning governance and accountability programme, which was aimed at ensuring that country-level commitments made at the 2012 London Summit on Family Planning and SRHR were met.

Q: Were they?

A: Some were, some weren’t. I mean, here we are in 2020, and I can tell you some progress has been made, but not nearly enough. There are several reasons for that, but one of them is the continued underfunding of basic PHC, including the vital promotive, preventive and non-health sector aspects I talked about. I honestly think we have been going backwards in some respects. I don’t know if the data bears this out but, anecdotally, that is my impression. Growing up in my village in the 1980s, I remember government people coming to talk to us about the importance of digging proper latrines, storing water safely, boiling water, growing kitchen gardens to ensure proper balanced nutrition. We were given guidance on all those things and we followed it. It seems to me that we have lost sight of this kind of comprehensive approach. These days discussions of health system strengthening and UHC – and believe me I take part in a lot of them – tend to focus on how many CT (computed tomography) scanners we have in how many facilities or how many hospitals we have in how many cities. Again, I am not saying we don’t need those things, but I think we could achieve so much more with the resources we have by following the Alma-Ata guidance regarding PHC, including those elements that fall outside the health sector. I mean can you imagine what we could achieve by investing in prevention and promotion and health literacy instead of building big expensive facilities? Certainly, our handling and experience of the ongoing pandemic would have been very different if we had established the foundation of basic PHC, including a health-literate, self-reliant population.

Q: Is the pandemic increasing awareness of the need for PHC in Kenya?

A: Certainly. First by exposing the lack of capacity to provide an adequate response to demand for treatment of severe cases of COVID-19 at health clinics, and second by exposing the lack of capacity to maintain essential PHC services, including maternal and child health and family planning services, during a surge in demand. Sexual activity does not stop during a pandemic and neither do pregnancies. We are seeing an increase in deaths of mothers at home and an increase in adolescent pregnancies, just two indicators of the toll the pandemic is taking, and which it would not be taking if we had a robust PHC system in place. So perhaps COVID-19 is providing an important wake-up call to our health system planners. Whether or not they are listening remains to be seen.

